# A Putative Transcription Factor MYT1 Is Required for Female Fertility in the Ascomycete *Gibberella zeae*

**DOI:** 10.1371/journal.pone.0025586

**Published:** 2011-10-03

**Authors:** Yang Lin, Hokyoung Son, Jungkwan Lee, Kyunghun Min, Gyung Ja Choi, Jin-Cheol Kim, Yin-Won Lee

**Affiliations:** 1 Department of Agricultural Biotechnology and Center for Fungal Pathogenesis, Seoul National University, Seoul, Korea; 2 Department of Applied Biology, Dong-A University, Busan, Korea; 3 Biological Function Research Team, Korea Research Institute of Chemical Technology, Daejeon, Korea; University of Wisconsin – Madison, United States of America

## Abstract

*Gibberella zeae* is an important pathogen of major cereal crops. The fungus produces ascospores that forcibly discharge from mature fruiting bodies, which serve as the primary inocula for disease epidemics. In this study, we characterized an insertional mutant Z39P105 with a defect in sexual development and identified a gene encoding a putative transcription factor designated as *MYT1*. This gene contains a Myb DNA-binding domain and is conserved in the subphylum Pezizomycotina of Ascomycota. The MYT1 protein fused with green fluorescence protein localized in nuclei, which supports its role as a transcriptional regulator. The *MYT1* deletion mutant showed similar phenotypes to the wild-type strain in vegetative growth, conidia production and germination, virulence, and mycotoxin production, but had defect in female fertility. A mutant overexpressing *MYT1* showed earlier germination, faster mycelia growth, and reduced mycotoxin production compared to the wild-type strain, suggesting that improper *MYT1* expression affects the expression of genes involved in the cell cycle and secondary metabolite production. This study is the first to characterize a transcription factor containing a Myb DNA-binding domain that is specific to sexual development in *G. zeae*.

## Introduction

The ascomycete fungus *Gibberella zeae* (anamorph: *Fusarium graminearum*) is an important plant pathogen that causes Fusarium head blight (FHB) in cereal crops and Fusarium ear and stalk rot in maize throughout the world [1]. The infection of this fungus leads to severe yield losses and accumulation of mycotoxins, such as trichothecenes and zearalenone, that are harmful to humans and livestock [2]. *G. zeae* produces sexual spores (ascospores) and asexual spores (conidia). Although both spores contribute to the disease propagation, ascospores that are forcibly discharged from mature fruiting bodies (perithecia) serve as the primary inocula for the epidemics of FHB in cereal crops [3], [4].

Perithecia or associated hyphae are known to be the components of fungi that aid in survival during the winter season [5], [6]. Sexual reproduction also helps the *G. zeae* population concurrently maintain genetic diversity and genetic stability [7], and a *G. zeae* population with reduced fertility has lower levels of genotypic diversity [8]. In addition, high genetic diversity of virulence-related genes is thought to provide *G. zeae* with a great capacity for adaptability toward host plants [9].

In ascomycetes, fruiting body formation requires the temporal and spatial control of cell differentiation, which is a process under polygenic control [10], [11]. To date, several genes and pathways have been reported to play important roles in the sexual development of *G. zeae* [12], [13], [14], [15], [16], [17], [18], [19], [20], [21], [22]. However, most of the genes characterized showed defects in the pleiotropic phenotypes, such as mycelial growth, conidiation, toxin production, virulence, and sexual development. Therefore, it would be valuable to identify and characterize genes that are specifically involved in perithecium formation for an in-depth understanding of sexual development. In particular, studies on transcription factors that are specific for sexual development will enable us to link upstream signaling pathways with downstream genes that have been previously characterized.

The Myb DNA-binding domain is typically found in eukaryotic transcription factors and has variable functions. The first identified protein containing the Myb DNA-binding domain was the v-Myb of the avian myelobastosis virus [23]. Three v-*myb* related genes, c-*myb*, A-*myb*, and B-*myb*, were subsequently found in several vertebrates [24]. Homologs were also identified in insects, fungi, and slime molds [25]. In animals, Myb-related proteins are known to be involved in the control of cell proliferation, apoptosis, and differentiation in particular hematopoietic progenitor cells [26], [27]. Moreover, the number of Myb genes is higher in plants than fungi or animals [28]. Bioinformatics analyses have identified 198 and 183 Myb genes in the *Arabidopsis* and rice genomes, respectively [29]. These proteins play important roles in various cellular processes [30].

In fungi, the roles of transcription factors containing the Myb DNA-binding domain largely remained unknown. *Schizosaccharomyces pombe* cdc5p was found to be essential for G2/M progression and pre-mRNA splicing [31]. Another Myb domain transcription factor, Reb1, was originally found to be involved in the termination of rRNA transcription. A recent study showed that Reb1 binds upstream of *ste9* ^+^ and is required for *ste9* ^+^ up-regulation and G1 arrest in response to nitrogen starvation. Consequently, the mating efficiency of cells lacking *reb1* was significantly reduced [32]. *Saccharomyces cerevisiae* BAS1 is known to be required for activation of GCN4-indepentdent *HIS4* transcription [33]. In filamentous fungi, only the FlbD homolog is known to control conidiophore development in *Aspergillus nidulans*, but has no identifiable role in *Neurospora crassa* [34], [35].

In this study, we identified a gene that encodes a putative transcription factor containing a Myb DNA-binding domain (*MYT1*) from *G. zeae* through restriction enzyme-mediated integration (REMI) mutagenesis. The mutant, Z39P105, in which the promoter region of *MYT1* gene had been disrupted, showed a defect in perithecia production, but not in other phenotypes. The objectives of this study were to determine whether the defect of Z39P105 was derived from *MYT1* disruption and to characterize the functions of the *MYT1* gene in *G. zeae*. The results demonstrate that *MYT1* plays an important role in perithecia development and is possibly involved in vegetative growth and toxin production.

## Methods

### Fungal strains and media

The wild-type strain GZ3639 [36] and mutants derived from this strain were stored as mycelia and conidia in 20% glycerol at −70°C. The mutant strain Z39P105 was generated by REMI [17]. A transgenic strain, mat1g, carrying both the *MAT1-1* deletion and histone H1 tagged with green fluoresce protein (GFP) derived from GZ3639 [37], was used for outcrosses to check the male and female fertility of *MYT1* deletion mutants. Another transgenic strain, mat1r [16], carrying both the *MAT1-1* deletion and histone H1 tagged with red fluoresce protein (RFP), was used in the co-localization study. All strains used in this study are listed in Table 1. Minimal medium containing 5 mM agmatine (MMA) was used for the trichothecene production [38]. Carboxymethylcellulose medium (CMC) [39] and yeast extract, malt extract agar (YMA) [40] were used for conidia production. Other media were prepared as described in the *Fusarium* laboratory manual [1].

**Table 1:** *G. zeae* strains used in this study.

Strain	Genotype	Source or reference
GZ3639	Wild-type	[36]
Z39P105	REMI mutant	This study
myt1	Δ*myt1::gen*	This study
MYT1com	Δ*myt1::MYT1-GFP-hyg*	This study
mat1g	Δ*mat1-1-1::gen*; *hH1::hH1-GFP-hyg*	[37]
MYT1OE	*MYT1::gen- P_EF1α_ -MYT1*	This study
MYT1OEG	*MYT1::gen- P_EF1α_ -GFP-MYT1*	This study
mat1r	Δ*mat1-1::gen*; *hH1::hH1-RFP-gen*	[16]
MYT1OEGr	*MYT1::gen- P_EF1α_ -GFP-MYT1*; *hH1::hH1-RFP-gen*	This study

### Nucleic acid manipulations, primers, and sequencing

Fungal genomic DNA was extracted according to the *Fusarium* laboratory manual [1]. Total RNA was isolated from mycelia or perithecia ground in liquid nitrogen using the Easy-Spin Total RNA Extraction Kit (Intron Biotech, Seongnam, Korea). Standard protocols were followed for restriction endonuclease digestion, agarose gel electrophoresis, and DNA gel blot hybridization with ^32^P labeled probes [41]. The PCR primers used in this study were synthesized at an oligonucleotide synthesis facility (Bionics, Seoul, Korea) (Table S1), diluted to 100 µM in sterilized water, and stored at −20°C. DNA sequencing was performed at the National Instrumentation Center for Environmental Management (Seoul National University, Seoul, Korea) and the sequences were compared against the *Fusarium* Comparative Database at the Broad Institute (http://www.broadinstitute.org/annotation/genome/fusarium_group/).

### Thermal asymmetric interlaced (TAIL) and rapid amplification of cDNA ends (RACE)-PCR

Thermal asymmetric interlaced (TAIL)-PCR was used to identify the plasmid insertion site of REMI mutant Z39P105 as previously described [42]. Six nested sequence-specific primers (PUCH1 5-1, PUCH1 5-2, PUCH1 5-3, PUCH1 3-1, PUCH1 3-2, and PUCH1 3-3) and shorter arbitrary degenerate primer sets (AD1, AD2, AD3, AD4, AD5, AD6, and AD7) were used to control the relative amplification efficiencies of specific and non-specific PCR products thermally. The DNA fragments adjacent to known plasmid sequences were recovered, purified using a GeneClean Turbo kit (Qbiogene, Carlsbad, CA, USA), and then directly sequenced.

The *MYT1* open reading frame (ORF) was determined by rapid amplification of cDNA ends (RACE)-PCR. The cDNA library was obtained from a previous study [16]. Three fragments located around the *MYT1* ORF were amplified with MYT1-seq1/MYT1-seq2, pPRN3-N-For/MYT1-seq2, and pPRN3-N-Rev/MYT1-seq1 primers and then directly sequenced.

### Genetic manipulations

A slightly modified double-joint (DJ) PCR strategy [43] was applied to construct fusion PCR products for targeted gene deletion. Firstly, the 5′ and 3′ flanking regions of *MYT1* were amplified from the wild-type strain using primer pairs MYT1-5F/MYT1-5R and MYT1-3F/MYT1-3R, respectively. Secondly, a geneticin resistance cassette (*gen*) under the control of the *A. nidulans trpC* promoter and terminator was amplified from pII99 [44] using the primer pair Gen-for/Gen-rev. Three amplicons (5′ flanking, 3′ flanking, and *gen*) were then fused by a second round of DJ PCR. Finally, a 4.9 kb DNA fragment was amplified with the nested primer pair MYT1-5N/MYT1-3N using a second round PCR product as template.

To complement the *MYT1* deletion mutant (Δ*myt1*), a fusion construct was generated by DJ PCR, which included the *MYT1* ORF with its own promoter, the green fluorescent protein gene (*GFP*), the hygromycin resistance gene cassette (*hyg*), and the 3′ flanking region of the *MYT1* gene. The *MYT1* ORF with its own promoter was amplified with the primer pair MYT1-5F/MYT1-5R GFP. The *GFP-hyg* was amplified from pIGPAPA [45], and the 3′ flanking region of the *MYT1* gene was amplified by primer pair MYT1-3F/MYT1-3R. The fusion construct was then transformed into the *MYT1* deletion mutant.

To construct mutants overexpressing *MYT1*, the 5′ flanking of *MYT1* and *MYT1* ORF were amplified by primer pairs MYT1-5F/MYT1-5R OE and MYT1-3F OE/MYT1-3R OE, respectively. A *gen-P_EF1α_* sequence, carrying elongation factor 1α promoter (*P_EF1α_*) from *Fusarium verticillioides*, was amplified from pSKGEN [46] with primers Neo-for new and eGFP-P1. Three amplicons were fused as described above. Using this fusion fragment as a template, a final PCR product was amplified by the nested primers MYT1-5N and MYT1-3N OE. The strain overexpressing a *GFP* tagged *MYT1* (MYT1OEG) was produced using the same strategy, and the only difference in strains was that the *gen* was followed by *P_EF1α_* and the *GFP* ORF.

### Quantitative real time (qRT)-PCR

Total RNA was isolated from vegetative cultures at 5 d after inoculation and at 3, 5, and 7 d after sexual induction using an Easy-Spin Total RNA Extraction Kit (Intron Biotech, Seongnam, Korea). The first strand cDNA was synthesized with SuperScriptIII reverse transcriptase (Invitrogen, Carlsbad, CA, USA). Quantitative real-time PCR (qRT-PCR) was performed by using SYBR Green Supermix (Bio-Rad, Hercules, CA, USA) and a 7500 real-time PCR system (Applied Biosystems, Foster City, CA, USA) with MYT1-realtime-F/MYT1-realtime-R primers (Table S1). The endogenous housekeeping gene cyclophilin (CyP1; locus ID: FGSG_07439.3) was used as an endogenous control for normalization [47]. The PCR was repeated three times with two replicates per run. The changes in fluorescence of the SYBR green dye in each cycle were monitored by the system software, and the threshold cycle (*C_T_*) above the background for each reaction was calculated. The gene expression was calibrated using the formula 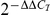 method as previously described [15]. The *C_T_* value of CyP1 was subtracted from that of *MYT1* to obtain a Δ*C_T_* value. The Δ*C_T_* value of *MYT1* expression in the wild-type vegetative stage at 5 d was subtracted from the Δ*C_T_* value of each sample to obtain a ΔΔ*C_T_* value. The *MYT1* transcript level relative to the calibrator was expressed as 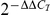. A Tukey test was conducted using SPSS 12.0 software (SPSS, Inc. Chicago, IL) to examine statistically significant differences (*p*<0.05) of 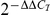 among the mean values of the samples.

### Fertility test

Mycelia grown on carrot agar for 5 d were mock fertilized to assess self-fertility as previously described [1]. For outcrosses, mycelia of the female strain grown on carrot agar plates were fertilized with 1 ml of male strain conidia suspension (1×10^6^ conidia ml^−1^), which was induced in CMC. The heterothallic mat1g (Δ*mat1-1*; *hH1-GFP*) strain [37] was used as a tester strain for outcrosses. Perithecia and ascospores were observed 9 d after fertilization.

### Conidia production and germination test

Each strain was incubated in 50 ml of complete media (CM) at 25°C on a rotary shaker (150 rpm). After 72 h, mycelia were harvested and washed twice with sterile distilled water. The mycelia were then spread onto YMA plates and incubated at 25°C for 48 h under near UV light (wavelength: 365 nm, HKiv Import & Export Co., Ltd., Xiamen, China) to induce conidiation. Conidia on the YMA were collected with sterile distilled water, filtered through cheese cloth, washed with sterile distilled water again, and then centrifuged (5000 rpm, 25°C, 5 min). The concentration of the conidia was adjusted to 10^5^ conidia ml^−1^ with distilled water. One ml of conidia suspension (1×10^5^ conidia ml^−1^) of each strain was inoculated into 50 ml CMC and incubated at 25°C on a rotary shaker (150 rpm).

The germination rate of conidia was measured as previously described [15]. In brief, 1 ml of harvested conidia from YMA medium was inoculated in 20 ml of CM and minimal medium (MM). The number of germinated conidia was counted after incubation at 0, 2, 4, 6, and 8 h. The experiment was performed twice with three replicates for each point.

### Virulence test and trichothecene analysis

For the virulence test, the point inoculation method was carried out as previously described [14]. The conidia suspension (10^5^ conidia ml^−1^) was prepared from CMC and then 10 µl of the conidia suspension from each strain was injected into a center spikelet of wheat head (cultivar; Eunpamil) midanthesis. After inoculation, wheat plants were incubated in a humidity chamber for 3 d and then transferred to a greenhouse. Spikelets with disease symptoms were counted 14 d after inoculation as previously described [22]. The experiment was performed with five replicate inoculations per strain, and two independent mutant strains were used for the experiment.

Trichothecene analysis was performed as previously described [16]. Briefly, MMA cultures were extracted with ethyl acetate, and the extracts were concentrated to dryness. A portion of each extract was derivatized with Sylon BZT (BSA+TMCS+TMSI, 3∶2∶3 respectively, Supelco, Bellefonte, PA, USA) and analyzed with a Shimadzu QP-5000 gas chromatograph mass spectrometer (GC-MS, Shimadzu, Kyoto, Japan) with a selected ion-monitoring mode as previously described [48]. Trichothecenes were quantified based on the biomasses produced by each strain. The experiment was repeated three times.

### Microscopic observation

To observe co-localization of MYT1 with nuclei, the mat1r strain [16] was fertilized with the MYT1OEG strain. Ascospores carrying both *P_EF1α_ -GFP-MYT1* and *hH1-RFP-gen* were selected using antibiotic resistance and confirmed by PCR. Localization was observed in cultures from CM, MM, and CMC.

Meiotic chromosomes were stained with acriflavin as previously described [49]. In brief, maturing perithecia on carrot agar were collected and hydrolyzed with 4 N HCl at 30°C for 20 min. After washing two times with distilled water, the samples were stained with acriflavin solution (100 µg acriflavin and 5 mg K_2_S_2_O in 1 ml of 0.1 N HCl) for 20 min. The stained samples were washed three times with washing solution (2 ml of glacial HCl and 98 ml of 70% ethanol [v/v]) and then washed twice with distilled water.

Microscopic observation was performed with a DE/Axio Imager A1 microscope (Carl Zeiss) using the filter set 38HE (excitation 470/40; emission 525/50) for GFP and acriflavin and the filter set 15 (excitation 546/12; emission 590) for RFP.

## Results

### Identification of MYT1

**Figure 1:**
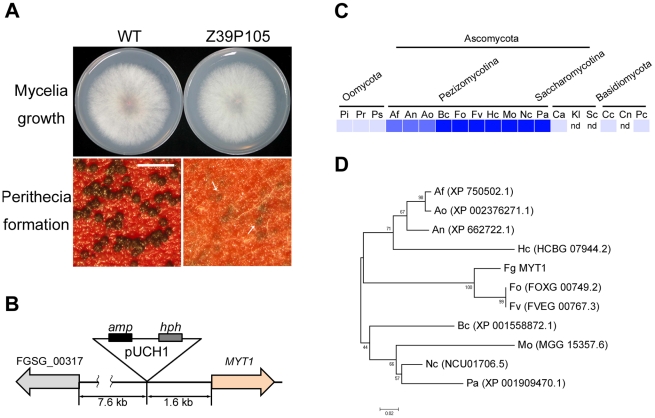
Identification of MYT1 and its distribution in fungi. (A) Mycelial growth and perithecium formation of the Z39P105 mutant on potato dextrose agar (PDA) and carrot agar, respectively. Pictures were taken 3 d after inoculation and 7 d after sexual induction from PDA and carrot agar. Arrows indicate protoperithecia. (B) Molecular characterization of the vector insertion event in the Z39P105 genome. (C) Distribution of MYT1 in representative fungal species. The distribution image was constructed by using the BLASTMatrix tool that is available on the Comparative Fungal Genomics Platform (http://cfgp.riceblast.snu.ac.kr/) [62]. (D) Phylogenetic tree of MYT1 homologs in several fungal species. The alignment was performed with ClustalW, and the MEGA program Version 4.0 was used to perform a 1,000 bootstrap phylogenetic analysis using the neighbor joining method. *amp*, ampicillin resistance gene; *hph*, hygromycin B resistance gene. Pi, *Phytophthora infestans*; Pr, *P. ramorum*; Ps, *P. sojae*; Af, *Aspergillus fumigatus*; An, *A. nidulans*; Ao, *A. oryzae*; Bc, *Botrytis cinerea*; Fo, *Fusarium oxysporum*; Fv, *F. verticillioides*; Hc, *Histoplasma capsulatum*; Mo, *Magnaporthe oryzae*; Nc, *Neurospora crassa*; Pa, *Podospora anserine*; Ca, *Candida albicans*; Kl, *Kluyveromyces lactis*; Sc, *Saccharomyces cerevisiae*; Cc, *Coprinus cinereus*; Cn, *Cryptococcus neoformans*; Pc, *Phanerochaete chrysosporium*; nd, not detected.

The REMI mutant strain Z39P105 produced a few protoperithecia without significant defects in vegetative growth (Figure 1A). Protoperithecia formed in the REMI mutant failed to differentiate further and there were no observable initial structures of asci or rosettes. Southern blot analysis was performed on Z39P105 gDNA digested with either *Bgl*II or *Kpn*I using the entire vector pUCH1 [50] as a probe and revealed a single insertion of the vector in the Z39P105 genome (data not shown). Using TAIL-PCR, we identified that the insertion site of pUCH1 was 1.6 kb upstream of FGSG_00318.3 and 7.6 kb upstream of FGSG_00317.3 (Figure 1B). Supposing that the promoter region of FGSG_00318.3 was disrupted in the Z39P105 mutant, we compared transcript level of this gene between wild-type strain and Z39P105 mutant and confirmed about 10-fold increased expression of FGSG_00318.3 in Z39P105 mutant in carrot agar (Figure S1). The result of RACE-PCR indicated that the transcription and splicing of FGSG_00318.3 *in vivo* were the same as the deduced ORF in the database. We designated FGSG_00318.3 as Myb DNA-binding domain containing transcription factor 1 (*MYT1*), which encodes a 294 amino acid polypeptide containing the Myb DNA-binding domain. MYT1 has no distinct homolog in the species in the phyla Oomycota and Basidiomycota, but is highly conserved in species of the subphylum Pezizomycotina of Ascomycota (Figure 1C, D). Deletion of the other candidate gene, FGSG_00317.3, which might be responsible for mutant phenotypes of Z39P105 mutant, did not show any defect in sexual development and the gene was excluded for this study (data not shown).

### Deletion, complementation, and overexpression

**Figure 2:**
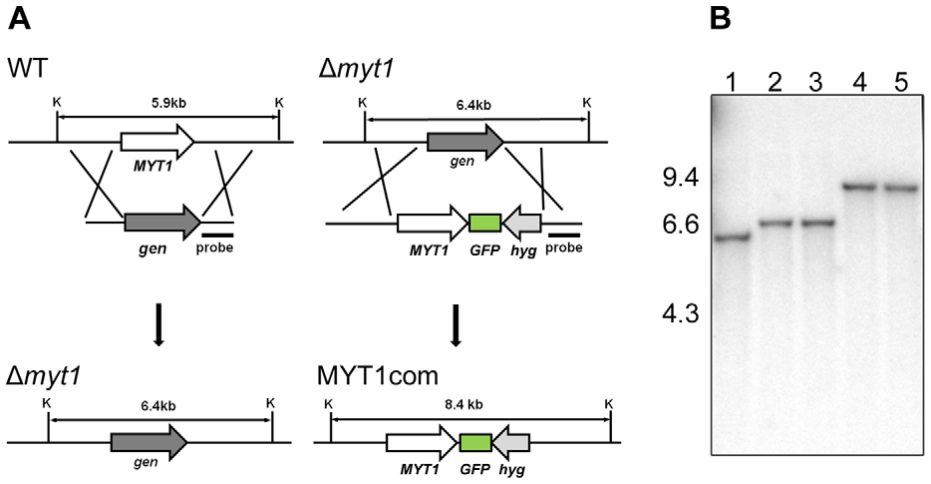
Targeted deletion and complementation of *MYT1*. WT, *G. zeae* wild-type strain GZ3639; Δ*myt1*, *MYT1* deletion mutant; MYT1com, Δ*myt1-*derived strain complemented with *MYT1*; K, *Kpn*I; *gen*, geneticin resistance gene cassette; *hyg*, hygromycin B resistance gene cassette. Lane 1, wild-type strain GZ3639; lane 2 and 3, deletion mutant; lane 4 and 5, complementation mutant. The sizes of DNA standards (kb) are indicated on the left of the blot.

**Figure 3:**
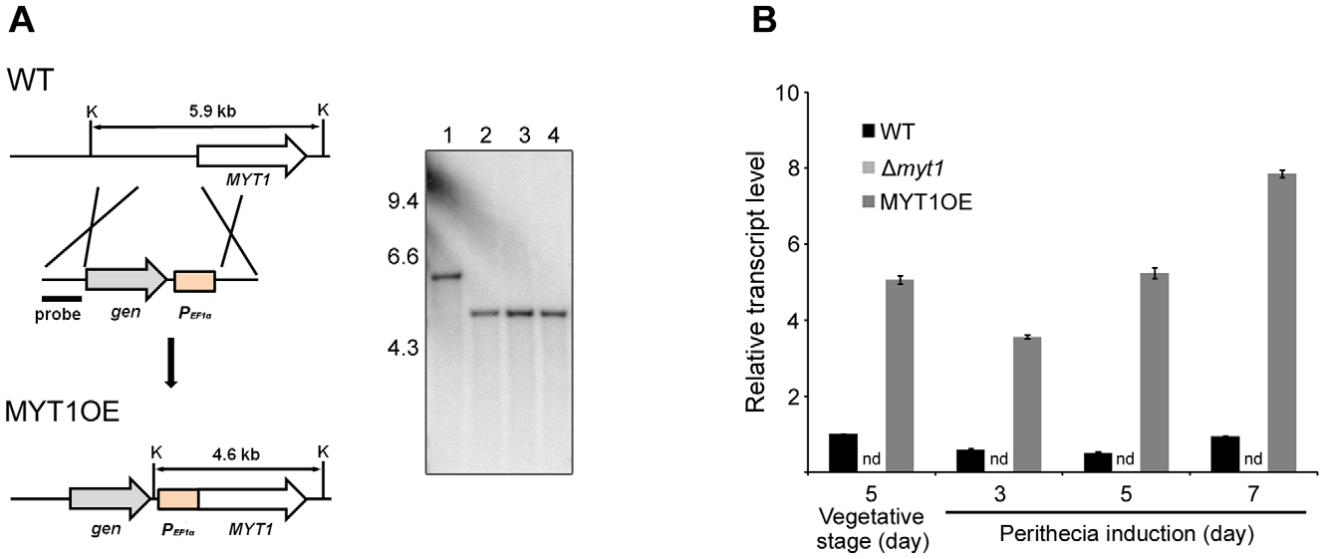
Overexpression of *MYT1*. (A) The *EF1α* promoter was inserted in the *MYT1* entire promoter region. The left and right panels show the strategy of MYT1OE strain construction and Southern hybridization, respectively. In the blot, lane 1 and 2–4 represent the wild-type strain and the *MYT1*-overexpressed mutant, respectively. (B) Expression of *MYT1* in wild-type, *MYT1*-deleted, and *MYT1*-overexpressed strains. Transcript level of *MYT1* was analyzed by quantitative real time-PCR (qRT-PCR) during the vegetative and sexual induction stages. WT, wild-type strain GZ3639; MYT1OE, transgenic strain where the *MYT1* promoter region was replaced with the *EF1α* promoter; K, *Kpn*I. The sizes of DNA standards (kb) are indicated on the left of the blot.

To investigate the function of *MYT1*, we performed targeted gene deletion and complementation. *MYT1* was successfully replaced with *gen* by homologous recombination and complemented with the GFP fusion construct (Figure 2). We also generated a MYT1OE strain in which *MYT1* is under control of the *EF1α* promoter (Figure 3A). All of the genetic manipulations were confirmed by Southern hybridizations (Figure 2 and Figure 3A).

To confirm the transcript level of the deletion and overexpression mutants, we performed qRT-PCR on wild-type, Δ*myt1*, and MYT1OE strains. *MYT1* was constitutively expressed during the vegetative stage and sexual development, but slightly reduced 3–5 d after sexual induction. The expression of *MYT1* in the MYT1OE strain was up-regulated compared to the wild-type strain during both the vegetative growth and sexual development, whereas *MYT1* was not expressed in the Δ*myt1* strain (Figure 3B).

### Sexual development and outcrosses

**Figure 4:**
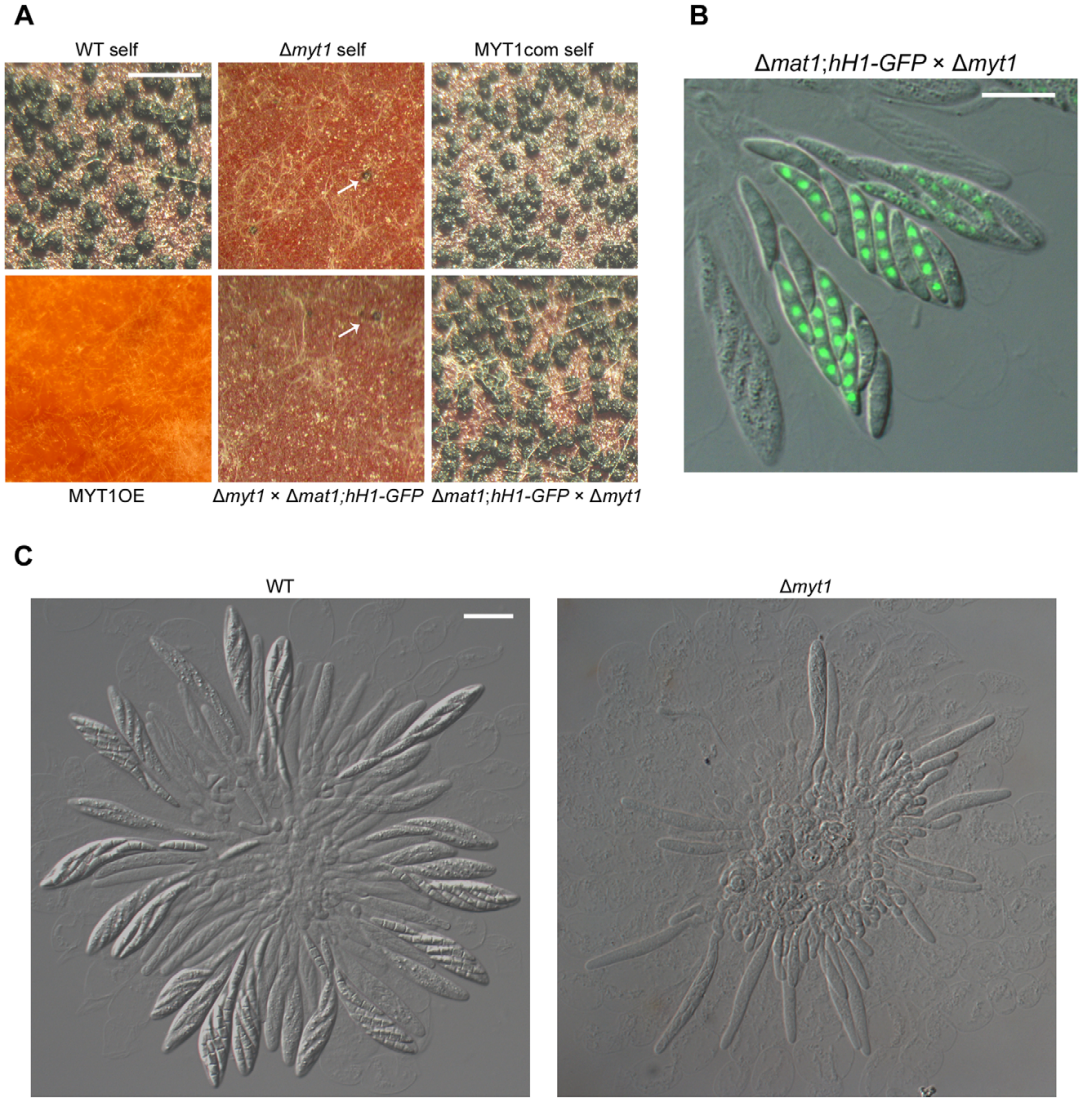
Sexual development of *MYT1* mutants. (A) Homozygous self-crosses and heterozygous outcrosses. Each strain was inoculated on carrot agar and was mock fertilized (self-cross) or outcrossed with male strain (Δ*mat1*; *hH1-GFP* and Δ*myt1* strains in Δ*myt1*×Δ*mat1*; *hH1-GFP* and Δ*mat1*; *hH1-GFP*×Δ*myt1*) outcrosses, respectively. The photographs were taken 10 d after sexual induction. The white arrows indicate immature perithecia. Scale bar = 1 mm. (B) Eight ascospores of an ascus from Δ*mat1*; *hH1-GFP*×Δ*myt1* outcross segregated into 1∶1 with and without GFP-tagged histone H1. Scale bar = 20 µm. (C) Morphology of asci rosettes. Microscopic observation was performed 8 d after sexual induction. Scale bar = 20 µm. WT, *G. zeae* wild-type strain GZ3639; Δ*myt1*, *MYT1* deletion mutant; MYT1com, Δ*myt1-*derived strain complemented with *MYT1*; MYT1OE, transgenic strain that has the *EF1α* promoter in place of the *MYT1* promoter region.

**Figure 5:**
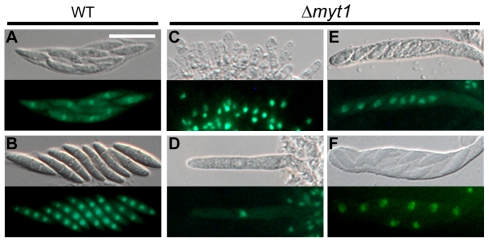
Meiotic chromosomes from selfings of wild-type strain (A and B) and MYT1 deletion mutant (C–F). Perithecia produced 8 d after sexual induction were strained with acriflavin. Scale bar = 20 µm.

Similar to the Z39P105 mutant, the self-cross of the Δ*myt1* strain resulted in more immature and fewer perithecia compared to the wild-type and complemented strains (Figure 4A). We performed outcrosses to check the female and male fertility of the Δ*myt1* mutant. When Δ*myt1* was fertilized as a female with conidia of wild-type or mat1g (Δ*mat1-1*; *hH1-GFP*) strains, only immature perithecia were found, similar to those formed in the self-cross of Δ*myt1*, indicating that Δ*myt1* lost female fertility. When the Δ*myt1* strain was used as a male for outcrossing of mat1g (female)×Δ*myt1* (male), normal perithecia were produced, and the progeny with or without the hH1-GFP signal segregated into 1∶1 in accordance with Mendelian genetics, suggesting that *MYT1* is not necessary for male fertility (Figure 4B). The mutant overexpressing *MYT1* did not produce any initial structures of perithecia when selfed or outcrossed as a female, but remained normal when outcrossed as a male (Figure 4A). When *MYT1* deletion mutant was selfed or outcrossed as female, ascus development was severely defective where most asci were arrested in crozier stage and even a few elongated asci did not contain normal ascospores (Figure 4C). Asci produced from the wild-type strain went through a normal meiotic process where each ascus contained eight nuclei and spore delimitation generated eight ascospores (Figure 5A and B). Meiotic chromosome staining revealed that the asci of *MYT1* deletion mutant were mostly in one nucleus stage (pachytene or metaphase-1) (Figure 5C and D). A few asci contained eight nuclei (Figure 5E) and began to delimit the ascospores (Figure 5F). However, the ascospores of *MYT1* deletion mutant did not mature further.

### Vegetative growth, conidiation, virulence, and trichothecenes production

**Figure 6:**
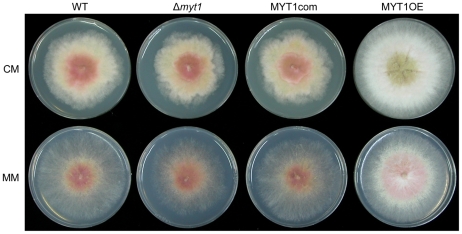
Vegetative growth of *MYT1* mutants. Each strain was grown on complete medium (CM) and minimal medium (MM) for 5 d. WT, *G. zeae* wild-type strain GZ3639; Δ*myt1*, *MYT1* deletion mutant; MYT1com, Δ*myt1-*derived strain complemented with *MYT1*; MYT1OE, transgenic strain that has the *EF1α* promoter in place of the *MYT1* promoter region.

**Figure 7:**
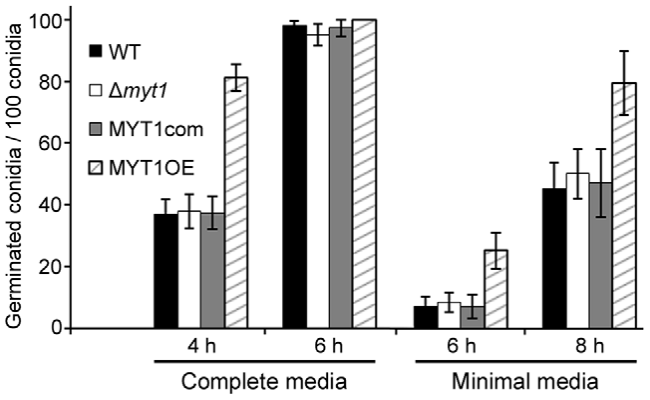
Germination rate of conidia. One ml of a conidia suspension of each strain was incubated in 10 ml of complete medium or minimal medium at 25°C on a rotary shaker (150 rpm). Two hundred spores were observed in each examination with light microscopy and the number of conidia that germinated was counted. All data were obtained from three biological replicates.

**Figure 8:**
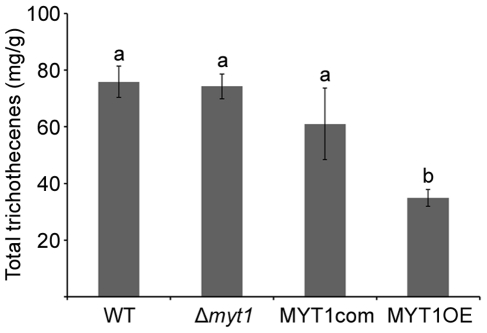
Total trichothecenes (deoxynivalenol and 15-acetyldeoxynivalenol) production by *G. zeae* strains. Each strain was grown in minimal medium supplemented with 5 mM agmatine for 7 d. Trichothecenes were analyzed by GC-MS and quantified based on the biomass produced by each strain. WT, *G. zeae* wild-type strain GZ3639; Δ*myt1*, *MYT1* deletion mutant; MYT1com, Δ*myt1-*derived strain complemented with *MYT1*; MYT1OE, transgenic strain that has the *EF1α* promoter inserted in place of the *MYT1* promoter region.

No differences in vegetative growth, conidia production, germination, virulence, and mycotoxin production were detected between the *MYT1* deletion mutant and the wild-type strain (Figure 6, 7, 8 and Figure S2). However, the MYT1OE mutant grew faster than the wild-type strain and accumulated less pigment (aurofusarin) when grown on CM and MM (Figure 6). In addition, conidia germination of MYT1OE was faster than that of other strains (Figure 7). Trichothecene production of the MYT1OE mutant was markedly reduced compared to other strains (Figure 8), but the virulence on wheat heads did not differ from the wild-type strain, even in the Δ*myt1* and MYT1OE strains (Figure S2).

### Localization of MYT1-GFP

**Figure 9:**
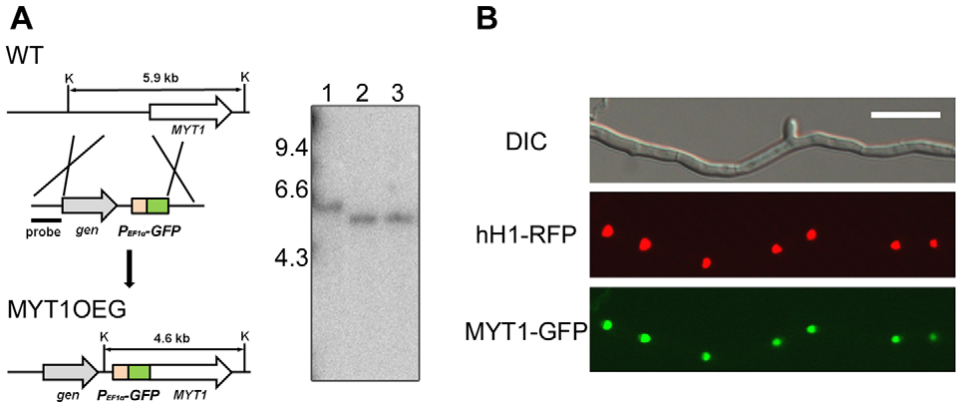
Cellular localization of MYT1. MYT1 was fused with green fluorescent protein (GFP) and histone H1 was fused with red fluorescent protein (RFP). (A) Strategy for GFP fusion with the *EF1α* promoter and (B) Co-localization of MYT1-GFP and hH1-RFP in mycelia. Scale bar = 20 µm.

The 16 *MYT1* complemented strains carrying the *MYT1-GFP* construct were confirmed by Southern hybridization, but none of them showed any detectable GFP signal in any of the observed fungal stages, including vegetative growth, conidiation, and sexual development. Since we hypothesized that *MYT1* expression was not strong enough for detection, we generated strains overexpressing the *MYT1* gene fused with *GFP* (MYT1OEG). We selected nine mutants carrying a single *GFP-MYT1* copy under the control of the *EF1α* promoter and observed a GFP signal in the nuclei of all nine strains. Phenotypes of MYT1OEG strains were similar with *MYT1* overexpression mutant (data not shown). To confirm nuclear localization of MYT1-GFP, MYT1OEGr (Δ*myt1::P_EF1α_ -GFP-MYT1-gen*; *hH1-RFP-gen*) was generated by performing an outcross between mat1r [16] and MYT1OEG. Localization was observed in cultures grown on CM and MM (for vegetative growth), YMA and CMC (for conidiation and conidia), and carrot agar (for sexual stage). In all of the stages, MYT1-GFP in the MYT1OEGr strain co-localized with hH1-RFP (Figure 9), which confirmed the nuclear localization of MYT1.

## Discussion

In this study, we identified and characterized a putative transcription factor MYT1, which has a specific function during sexual development in *G. zeae*. In-depth phenotyping revealed that the *MYT1* deletion mutant is female sterile, but is normal in male fertility and in other biological processes, including vegetative growth, conidiation, toxin production, and virulence. Since MYT1 contains the Myb DNA-binding domain and is exclusively localized in nuclei, MYT1 might have a pivotal regulatory role in the sexual reproduction and may participate in activation or repression of genes required for cell proliferation and differentiation in *G. zeae*. In addition, conservation of MYT1 in fungal species of the subphylum Pezizomycotina of Ascomycota suggests a conserved role during fruiting body formation.

Members of the Myb gene family have diverse roles as transcriptional regulators for multiple cellular processes in animals and plants, including cell proliferation, apoptosis, differentiation, metabolic pathways, cell fate and identity, and stress responses [26], [27], [30], [51], [52], [53], [54]. In fungi, the role of Myb-domain containing transcription factors that have been characterized play important roles in cell differentiation and proliferation, even though Myb family proteins show functional diversity [31], [33]. Our results also showed that MYT1 is involved in cell differentiation during sexual reproduction in *G. zeae*.

MYT1 was found to be constitutively expressed from the vegetative stage to sexual reproduction, even though the transcript level was not sufficient for detection by Northern hybridization (data not shown). The transcript level of *MYT1* in the wild-type strain was down-regulated at the start of sexual development and recovered as time progressed. Many genes related to sexual development are highly expressed from the beginning of sexual induction and increase expression as perithecia mature [55], [56]. Several genes required for both sexual development and vegetative growth, such as *FBP1* [17], *GzSNF1* [15], *ACL1*, and *ACL2* [16], are also highly expressed during the vegetative growth stage, decline immediately after sexual induction, and then increase again, similar to *MYT1* expression. The expression pattern of *MYT1* suggests a possible role during this growth period similar to other genes involved in both perithecium formation and mycelia growth.

Overexpression of *MYT1* resulted in faster mycelial growth and earlier germination but less production of secondary metabolites (aurofusarin and trichothecenes) than the wild-type strain although deletion of *MYT1* did not alter those phenotypes. In addition, *MYT1* overexpression mutants, REMI mutant and MYT1OE, showed more severe defects in sexual development than MYT1 deletion mutant. This abnormality might be derived from the improper expression of genes regulated by the transcription factor or overexpression of *MYT1* may affect the expression of other non-target genes. In many cases, the overexpression of certain genes triggers an unexpected phenotype changes in filamentous fungus. For example, the *A. nidulans abaA* gene encodes a transcription factor containing an ATTS DNA-binding motif and is required for the terminal stages of conidiophore development. The *abaA* mutant fails to form any viable conidia, with morphologically normal phialide production [57]. However, overexpression of *abaA* does not lead to spore formation, but it strongly inhibits growth and causes major morphological changes [57]. Increased transcript level of *MYT1* in REMI mutant suggests that vector insertion region of the REMI mutant might be closely related to transcriptional regulation of the gene and further promoter analysis will be helpful to reveal the regulatory mechanism of *MYT1*.

MYT1 is important for ascospore maturation even though the other sexual reproduction stages were also related to MYT1. In-depth microscopic observation revealed that in most cases ascus development of *MYT1* deletion mutant was stopped at the early stage or markedly delayed and asci did not produce mature ascospores, suggesting that deletion of *MYT1* suppressed ascus development and halted further ascospore maturation.

It has been reported that the extent of female sterility within a population varies widely by population. One study demonstrated that the relative amounts of sexual and asexual reproduction occurring in a population can be estimated based on relative frequency of the female-fertile strains [58]. The prerequisite of this theory is the presence of numerous loci where mutations can occur naturally and result in female sterility. It is likely that female sterility mutations that accumulate in field populations are specific for the sexual portion of the life cycle and have few, if any, effects on vegetative growth and sporulation. In recent years, several genes and pathways have been reported to play important roles in female fertility, such as mating type genes [59], [60] as well as G-protein and MAP-kinase signaling pathways [18], [19], [20], [61]. A novel b-ZIP transcription factor, ZIF1, has also been shown to play an important role in female fertility of *G. zeae* [22]. However, deletions of these genes or pathways are insufficient to explain the widespread female sterility observed in many field populations of *Fusarium* species, since there are multiple functions of these genes and pathways throughout the fungal life cycle. Thus, female sterile field stains might have defects in sexual specific genes, such as *MYT1*.

In conclusion, we report that MYT1 is required for female fertility in *G. zeae* and predict that MYT1 is involved in cell differentiation and proliferation during perithecium formation and possibly during vegetative growth. To our knowledge, this study is the first report characterizing a protein that contains a Myb DNA-binding domain, with the exception of FlbD homologs in filamentous fungi.

## Supporting Information

Figure S1 **Expression of** ***MYT1*** **in the wild-type and REMI mutant strains.** Transcript level of *MYT1* was analyzed by quantitative real time-PCR (qRT-PCR) during the vegetative growth on carrot agar. WT, wild-type strain GZ3639; Z39P105, REMI mutant.
(TIF)

Figure S2 **Virulence of** ***G. zeae*** **strains on wheat heads.** A center spikelet of each wheat head was injected with 10 µl of conidia suspension. Mock, negative control mock-inoculated with 0.01% of Tween 20; WT, *G. zeae* wild-type strain GZ3639; Δ*myt1*, *MYT1* deletion mutant; MYT1com, Δ*myt1-*derived strain complemented with *MYT1*; MYT1OE, transgenic strain that has the *EF1α* promoter inserted in place of the *MYT1* promoter region. The photographs were taken 14 d after inoculation.
(TIF)

Table S1 **Primers used in this study.**
(PDF)
