# Supplementary figures and images for: A Putative Transcription Factor MYT1 Is Required for Female Fertility in the Ascomycete Gibberella zeae
Source: PLoS One. 2011 Oct 3;6(10):e25586. doi: 10.1371/journal.pone.0025586 (PMC3184970; doi:10.1371/journal.pone.0025586)

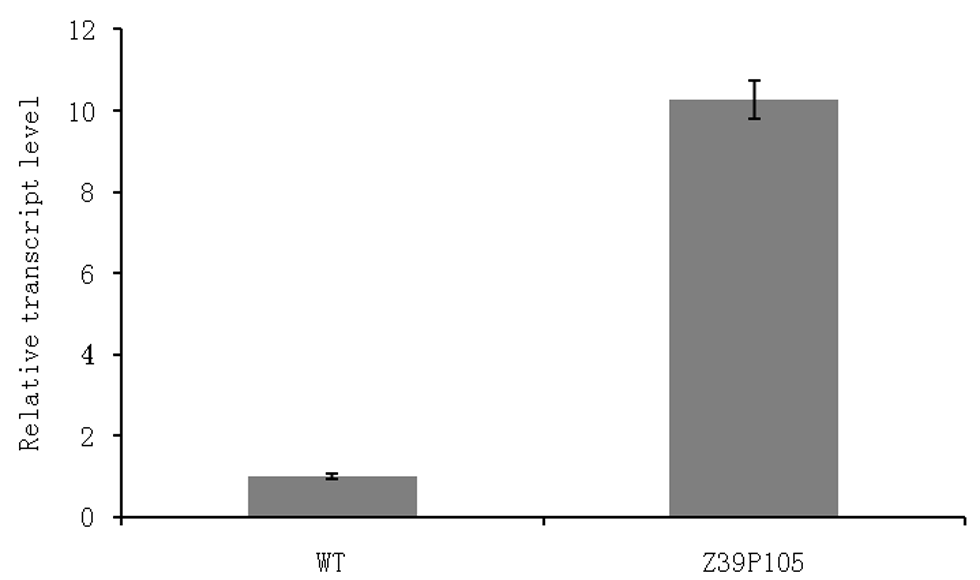

Supplement: Figure S1 — Expression of MYT1 in the wild-type and REMI mutant strains. Transcript level of MYT1 was analyzed by quantitative real time-PCR (qRT-PCR) during the vegetative growth on carrot agar. WT, wild-type strain GZ3639; Z39P105, REMI mutant. (TIF) [file pone.0025586.s001.tif]

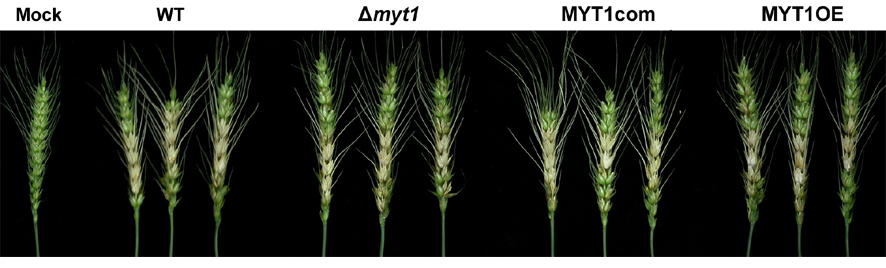

Supplement: Figure S2 — Virulence of G. zeae strains on wheat heads. A center spikelet of each wheat head was injected with 10 µl of conidia suspension. Mock, negative control mock-inoculated with 0.01% of Tween 20; WT, G. zeae wild-type strain GZ3639; Δmyt1, MYT1 deletion mutant; MYT1com, Δmyt1-derived strain complemented with MYT1; MYT1OE, transgenic strain that has the EF1α promoter inserted in place of the MYT1 promoter region. The photographs were taken 14 d after inoculation. (TIF) [file pone.0025586.s002.tif]
