# Supplementary material for: A Putative Transcription Factor MYT1 Is Required for Female Fertility in the Ascomycete Gibberella zeae
Source: PLoS One. 2011 Oct 3;6(10):e25586. doi: 10.1371/journal.pone.0025586 (PMC3184970; doi:10.1371/journal.pone.0025586)
Supplement: Table S1 — Primers used in this study. (PDF) [file pone.0025586.s003.pdf]

Table S1. Primers used in this study

Primer	Sequence(5'→3')	Description
PUCH1 5-1	GCTGCTGCATTCCCATTCCCATC	Specific primers used for TAIL-PCR that are complementary to the pUCH1 vector. The inner primers (pUCH1 5-2, pUCH1 3-2) and the innermost primers (pUCH1 5-3, pUCH1 3-3) were used to facilitate the confirmation of product specificity.
PUCH1 5-2	TCCCATCGTGGTCGAGCTACAAGA	
PUCH1 5-3	GCGCAGGTGGGCCTTGACAT	
PUCH1 3-1	AATGCAGCTGGCACGACAGGTTTC	
PUCH1 3-2	CACTCATTAGGCACCCCAGGCTTT	
PUCH1 3-3	TGTTGTGTGGAATTGTGAGCGGAT	
AD1	TG(A/T)GNAG(A/T)ANCA(G/C)AGA	Shorter arbitrary degenerate primers for controlling the relative amplification efficiencies of specific and non specific PCR products thermally
AD2	(G/C)TTGNTA(G/C)TNCTNTGC	
AD3	(A/T)CAGNTG(A/T)TNGTNCTG	
AD4	(A/T)AGTGNAG(A/T)ANCANAGA	
AD5	(A/T)AGTGNAG(A/T)ANCANGTT	
AD6	(A/T)AGTGNAG(A/T)ANCANGAA	
AD7	AG(A/T)GNAG(A/T)ANCA(A/T)AGG	
MYT1-5F	TCGAAGGCGAACAAAGGAAGACT	Forward and reverse primers for amplification of 5' flanking region of *MYT1* with tail for geneticin resistance gene cassette fusion
MYT1-5R	GCACAGGTACACTTGTTTAGAGTGCTCTGTGTCCGTTCCTTGTTC	
MYT1-3F	CCTTCAATATCATCTTCTGTCGTGAACGCTCGAGCAATTGTATCAC	Forward and reverse primers for amplification of 3' flanking region of *MYT1* with tail for geneticin resistance gene cassette fusion
MYT1-3R	AAAACGGAGCCGCCTAAACAAG	
MYT1-5N	GGGCGAACAAAGATGGGGACAAT	Forward and reverse nest primers for third fusion PCR for amplification of *MYT1* deletion construct
MYT1-3N	GCTACCCACGTCCACGCAGTCTAT	
MYT1-seq1	CACAGCCAGTTCCAGCTTTCACTTAT	For RACE-PCR of *MYT1*
MYT1-seq2	TGGTGGCGCTCTGTTATACACAACT	
pPRN3-N-For	GTCGAAAATTCAAGACAAGG	For RACE-PCR
pPRN3-N-Rev	AAGCGTGACATAACTAATTAC	For RACE-PCR

FGSG_00317-5F	CGTTTGGACACTACCAGCCCTATC	Forward and reverse primers for amplification of 5' flanking region of FGSG_00317 with tail for geneticin resistance gene cassette fusion
FGSG_00317-5R	GCACAGGTACACTTGTTTAGAGGTCGATAGATTGGCGGGGTATGT	
FGSG_00317-3F	CCTTCAATATCATCTTCTGTCGGAGGGGAAATGCAGGGTAGATGAT	Forward and reverse primers for amplification of 3' flanking region of FGSG_00317 with tail for geneticin resistance gene cassette fusion
FGSG_00317-3R	CGGCAGTGAAAGGTGATGTATTGAG	
FGSG_00317-5N	CAGACCCGGACTGAAGGAAGTTACG	Forward and reverse nest primers for third fusion PCR for amplification of FGSG_00317 deletion construct
FGSG_00317-3N	CAACGAAACGGTTATATCGCACTGAAC	
MYT1-5R GFP	GAACAGCTCCTCGCCCTTGCTCACTGCGATGCGTCGTCGTTGAT	Reverse primer for amplification of 5’ flanking region and ORF of *MYT1* with tail for *gfp* tagging complementation
MYT1-5R OE	GATAGTGGAAACCGACGCCCCTGCTCTGTGTCCGTTCCTTGTTC	Reverse primer for amplification of 5’flanking region of *MYT1* with tail for *gen* tagging overexpression
MYT1-3F OE	CGGCATGGACGAGCTGTACAAGATGTCTGGAAGGCGAGGTATG	Forward primer for amplification of *MYT1* ORF with tail for *EF promoter* tagging overexpression
MYT1-3R OE	GTGGCACGTTCCAGCGATACT	Reverse primer for amplification of *MYT1* ORF for overexpression
MYT1-3N OE	AAACAAAGACAAAGCGCTGTCAACTG	Reverse nest primer for third fusion PCR for amplification of *MYT1* overexpression construct
MYT1-3F GFPOE	TATCACAAAAGGAACCCAATCTTCAAAACACAGATGTCTGGAAGGCGAGGTAT	Forward primer for amplification of *MYT1* ORF with tail for *gfp* tagging overexpression
MYT1-realtime-F	CCCTTGTATTCAAGTCTCGCCAGTT	For realtime-PCR of MYT1
MYT1-realtime-R	GTCGTCGTTGATTTGGCTCGTATT	
Cyp1-realtime-F	TCAAGCTCAAGCACACCAAGAAGG	For realtime-PCR of Cyp1
Cyp1-realtime-R	GGTCCGCCGCTCCAGTCT	
